# Has the world ceased to care about HIV? And what can we do about it?

**DOI:** 10.7448/IAS.18.1.20818

**Published:** 2015-12-01

**Authors:** Mark A Wainberg, Susan Kippax, Marlene Bras, Papa Salif Sow

**Affiliations:** 1 McGill University AIDS Centre, Jewish General Hospital, Montreal, QC, Canada; 2 Journal of the International AIDS Society, Geneva, Switzerland; 3 Social Policy Research Centre, University of New South Wales, Sydney, Australia; 4 Department of Infectious Diseases, University of Dakar, Dakar, Senegal

Same time the past year, the world had been transfixed by the Ebola crisis that thankfully now seems to have largely disappeared. In contrast, in recent months, the world's attention has been focussed on the refugee crisis, and, indeed, it is easy to identify with the plight of so many desperate people who are anxious to escape the turmoil of their countries in the Middle East. Currently, climate change is on the political agendas of most if not all nations. These issues, although very different from one another, present huge challenges to our world and the lives of people who inhabit it. Global solidarity is urgently needed to solve these crises.

Sadly though, it seems that issues related to HIV/AIDS no longer figure on the world's list of priorities, even though the HIV epidemic continues to claim numerous lives on a daily basis. It is estimated that more than four million people will die of HIV/AIDS this year, while an equal number of individuals are likely to be newly infected.

Is it simply that HIV has been around for too long and that people now take it for granted? Is it because too many people perceive that the substantial progress achieved with regard to HIV treatment in the form of antiretroviral (ARV) drug development and access to these drugs does not make it much of a problem? Is it that too many people are unaware that the very successful drug rollout programs for HIV that have been spearheaded by the World Health Organization, UNAIDS and other international bodies have spurred increased levels of HIV drug resistance in virtually all developing countries even though the successful use of these very same drugs has enabled millions of people to survive HIV? What about the sub-standard therapies and second-tier diagnostics that are already the norm in so many developing countries in sub-Saharan Africa? And what has happened to the HIV-prevention programs, including condom promotion and the provision of opioid substitution therapy and sterile needles and syringes for injection drug users? Although the ARV drugs offer huge potential with regard to the prevention of new infections, many countries cannot afford them. Why are the rates of new HIV infections rising in some communities in the income-rich world? What of community engagement in HIV prevention and treatment?

Journalists seem so eager to focus on new problems that they forget about existing ones that represent as big a threat to the survival of millions of people. Is it because any new challenging situation will always represent a better opportunity to make the headlines rather than focusing on old tired stories that have ceased to have the attraction of novelty? The same fate has overtaken equally devastating diseases such as Malaria which also accounts for millions of lives on an annual basis. However, thankfully, well-deserving scientists were awarded the Nobel Prize in Medicine this year for their ground-breaking research on Malaria, carried out in some cases more than 30 years ago [1].

There is a lot to be said though about the wonderful progress that has been accomplished in the field of HIV over the past year. Several examples are as follows:

1. New WHO guidelines recommend initiation of antiretroviral therapy for all HIV-positive persons as early as possible, regardless of their CD4 level [2];
2. New data provide evidence for the efficiency of pre-exposure prophylaxis (PrEP) as an important prevention tool [2];
3. New and promising antiretroviral drugs, including novel anti-integrase compounds, are increasingly becoming available for treatment, and some of these, such as dolutegravir, seem to have the best profile of all antiretroviral therapies with regard to non-development of drug resistance [3];
4. New approaches are available for the use of long-acting antiretroviral therapies that have been formulated as injectables for both prevention and treatment [4].

But many challenges remain, such as:

1. How can we facilitate and improve access to and take-up of HIV testing, which is the entry point for treatment? As an example, only 45% of people in sub-Saharan African know of their HIV status [5];
2. How can we improve access to viral load point-of-care tests, which remain unavailable in many HIV high burden countries although they are essential to monitor and to avoid the occurrence of HIV drug resistance;
3. How can we simplify the journey of the HIV patient to avoid loss-to-follow-up and limited retention to therapy, in order to increase the number of people living with HIV who are virally suppressed?
4. Can we progress towards both preventive and therapeutic HIV vaccines?
5. In the absence of a preventive vaccine, how can we reinvigorate the earlier and very successful prevention interventions, including HIV-prevention education in schools, community engagement focussing on condom protection and other behavioural strategies, and the provision of opioid substitution therapy and sterile needles and syringes for injection drug users?

Our ultimate goal must be to improve prevention and treatment, reduce stigma and increase numbers of people living with HIV who are virally suppressed. While we strive to end the HIV epidemic, we must not forget that these challenges require more research, more money and generally more public awareness.

The XIII International AIDS Conference held in Durban, South Africa, in 2000 was one of the landmark AIDS conferences in the world. Community members and journalists have played a crucial role in ensuring access to HIV drugs becomes a priority of the political leaders’ agenda and a reality for infected people all over. At the time, HIV was considered a news-worthy topic for journalists. Unfortunately, this is no longer the case and we must find a way of once again making it so. How can we ensure that the threat of HIV and AIDS evokes the same emotional responses in people as other challenging world situations and thereby inspire people to action?

Clearly, it is imperative that the world offer a more united voice with regard to dealing with problems that afflict people everywhere, and this must also include HIV. We are still far from annihilating the HIV epidemic, and millions of people are dying of AIDS with millions being newly infected. What might we do to better remedy this situation? The world's initial very successful response is in need of reinvigoration.
